# Quantification of vestibular-induced eye movements in zebrafish larvae

**DOI:** 10.1186/1471-2202-11-110

**Published:** 2010-09-03

**Authors:** Weike Mo, Fangyi Chen, Alex Nechiporuk, Teresa Nicolson

**Affiliations:** 1 Howard Hughes Medical Institute, Oregon Hearing Research Center and Vollum Institute, Oregon Health and Science University, 3181 SW Sam Jackson Park Road, Portland, OR 97239, USA; 2 Oregon Hearing Research Center, Oregon Health and Science University, 3181 SW Sam Jackson Park Road, Portland, OR 97239, USA; 3 Department of Cellular and Developmental Biology, 3181 SW Sam Jackson Park Road, Oregon Health and Science University, Portland, OR 97239, USA

## Abstract

**Background:**

Vestibular reflexes coordinate movements or sensory input with changes in body or head position. Vestibular-evoked responses that involve the extraocular muscles include the vestibulo-ocular reflex (VOR), a compensatory eye movement to stabilize retinal images. Although an angular VOR attributable to semicircular canal stimulation was reported to be absent in free-swimming zebrafish larvae, recent studies reveal that vestibular-induced eye movements can be evoked in zebrafish larvae by both static tilts and dynamic rotations that tilt the head with respect to gravity.

**Results:**

We have determined herein the basis of sensitivity of the larval eye movements with respect to vestibular stimulus, developmental stage, and sensory receptors of the inner ear. For our experiments, video recordings of larvae rotated sinusoidally at 0.25 Hz were analyzed to quantitate eye movements under infrared illumination. We observed a robust response that appeared as early as 72 hours post fertilization (hpf), which increased in amplitude over time. Unlike rotation about an earth horizontal axis, rotation about an earth vertical axis at 0.25 Hz did not evoke eye movements. Moreover, vestibular-induced responses were absent in mutant *cdh23 *larvae and larvae lacking anterior otoliths.

**Conclusions:**

Our results provide evidence for a functional vestibulo-oculomotor circuit in 72 hpf zebrafish larvae that relies upon sensory input from anterior/utricular otolith organs.

## Background

Vestibular-induced behaviors can be used to measure vestibular function. For example, the VOR is a simple reflex of eye movements used for assessment of semicircular canal function in human patients [1]. This robust reflex has also been used to assess vestibular function in several different species, including monkeys [2] and rats [3]. The VOR is characterized by compensatory eye movements in response to linear or angular accelerations, and to any changes in head position with respect to gravity. Rotation around the earth vertical axis is sensed by the semicircular canal system, which generates an angular VOR. Linear or translational acceleration drives the linear VOR, which is activated by macular organs with otoliths or otoconia. Rotations about an off-vertical axis also stimulate mainly otolith organs if performed at a constant speed [4]. In addition to responses to dynamic stimuli, a roll-induced static tilt VOR, which is a static eye-to-head position change, has been characterized in many species including tadpoles [5] and fish larvae [6].

The vestibular system is highly conserved among vertebrates with respect to both anatomy and the genes required for function of the inner ear [7]. The inner ear of zebrafish larvae contains three developing semicircular canals, as well as anterior and posterior maculae destined to become the utricular and saccular organs, respectively. Due to the imaging techniques and genetic tools available, the zebrafish is emerging as a popular model for studies of neurobiology and behavior, including the molecular basis of auditory and vestibular function. Many mutations affecting hearing and balance to differing degrees have been identified in zebrafish [8,9], giving rise to the need to quantitate auditory/vestibular function in larvae. Analysis of vestibular-induced behavior in larvae is useful for determining the severity of balance deficits in auditory/vestibular mutants, and may ultimately yield insight into the in vivo function of the gene products.

Studies of vestibular-induced eye movements in zebrafish describe either static or dynamic responses. A static, linear VOR was examined by tilting the head down in embryonic or larval fish and observing changes in the angle of the eyes [10]. The resting eye angle was dependent upon the presence of the anterior otolith during development. Experiments focusing on the dynamic VOR in zebrafish larvae have yielded conflicting results. Easter and Nicola examined the development of several behaviors in zebrafish including the optokinetic response (OKR), as well as the angular VOR, and reported that both reflexes were present at 3 days post fertilization (dpf) [11]. In a later study, Beck et al. used a microscopic system with infrared illumination to measure the angular VOR, and found that zebrafish did not have an angular VOR until 35 dpf [12]. The authors suggested that the angular VOR of larval zebrafish observed previously was probably due to the OKR, in which eye movements are driven by changes in visual cues.

In two recent studies, a larval response to vestibular stimulation was detectable upon rotation about an earth horizontal axis [13,14]. We sought to expand on these findings by exploring the effects of varying the experimental parameters and genetic backgrounds of larvae on vestibular-induced eye movements. We confirmed that the larval response was vestibular and not visual, and we determined the ontogeny of the vestibular-evoked response. Our experiments provide evidence that the anterior otolith is required for sensing changes in linear acceleration and evoking the VOR observed during rotations in the vertical plane.

## Methods

### Animals

Animals used in this study were wild-type zebrafish larvae in the Tübingen or long fin background, and mutants identified in the present study (*rock solo^AN66^*) or previous studies (*cdh23^1619ag ^*and *synj1^Q296X^*; [14-16]). The *rock solo *mutant (recessive lesion) was identified from an ethylnitrosourea mutagenesis screen using a Tübingen background. Fish embryos and larvae were kept at 30°C in E3 embryo medium [17]. If necessary, 20 μl pronase was added into the medium to help larvae hatch out of the chorion at 2 dpf, followed by a change of E3 medium. All of the behavioral tests were carried out at room temperature (22-25°C).

**Figure 1:**
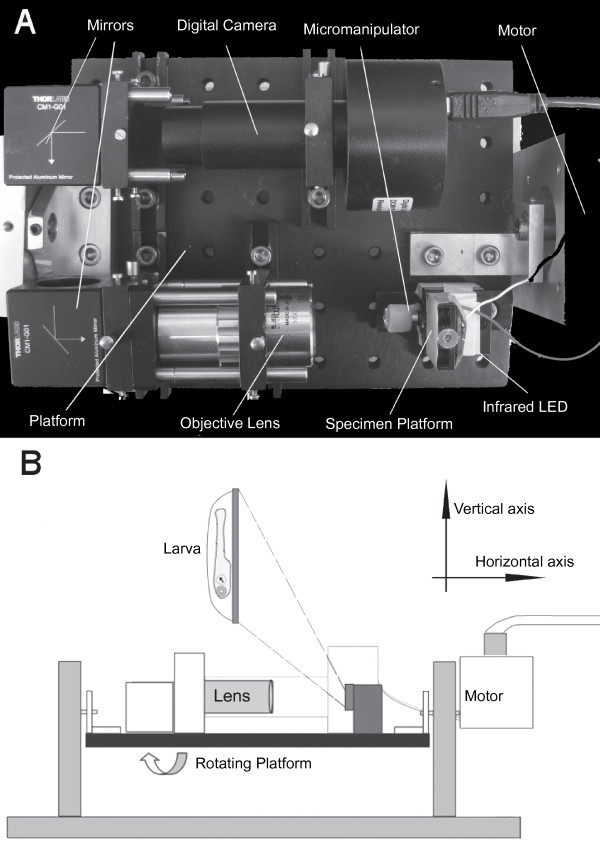
**The equipment and diagram of the experimental set up used to evoke eye movements in zebrafish larvae**. (A) An overview of the device constructed to stimulate and record vestibular-induced eye movements in larvae. (B) An illustration of the set up. Larvae were mounted on the specimen platform in a head-down position perpendicular to the platform. The platform was rotated around the axis shown by the curved arrow. The coordinate indicates the axes of two type of rotations used in this study.

We performed our experiments with 3-5 day old zebrafish larvae. For mounting, 2% low melting agarose in E3 media was kept at 42°C in a heating block. To immobilize fish larvae, a drop of low melting agarose was put on a cover slip and a larva was transferred into the agarose liquid by a glass pipette with minimal E3 media. Then the larva was adjusted to a dorsal-up position using fine forceps before the agarose solidified. The cover slip was placed on a metal rack for 5 minutes to allow the agarose to become firm. In order to free the eyes, a 0.5-1 μl region was excavated around the fish head using fine forceps, and then the exposed area was filled with E3 media. The E3 media allowed the eyes to move freely. The cover slip was then put onto the specimen platform, on which the larva was positioned head down, perpendicular to the platform plane (Figure 1).

### Microscopic system

A customized microscopic system was constructed to monitor the eye movements during rotation. As shown in Figure 1A, this system was composed of a Mitutoyo 5 × long working distance lens and a digital eyepiece (DCM300; Hangzhou Scopetek Opto-Electric, Zhejiang, China). Two 45° mirrors were placed between the objective and the digital eyepiece to guide the light. The U-shaped setup makes efficient usage of the rotation plate space and balances the motor load. All components were mounted on an aluminum platform, which could be rotated by a motor system with the supporting structure. Since all of the parts were fixed on the platform, no relative motion existed between the specimen and the eyepiece during the rotation process. This guaranteed a consistent viewing area during the experiment, which avoided blurring due to the relative motion between the camera and the fish. The same area under constant illumination also warranted relatively constant image brightness on each frame during a single trial.

A servo motor (Model# BE231DJ-NPSN, Parker Hannifin, Cleveland, OH, USA) and servo controller (Model# GV6K-U3E, Parker Hannifin) were used to rotate the platform, which held the microscopic system. A motor gear head (Model # 23SP100, Parker Hannifin) with a gear ratio of 1:100 was attached to the servo motor to reduce the speed and increase the torque. This servo motor system can control the angular position of the platform with a precision of less than 0.2 degree. An Ethernet cable connects the controller and a computer, allowing the computer to program the motor rotation profile and read the motor position.

All experiments were conducted in the dark with a cover box, if not otherwise specified. Larvae were mounted on a transparent specimen plate, which was supported by a 3 D micromanipulator. Each fish was trans-illuminated by an infrared LED with emission wavelength around 820 nm. A dark background with infrared illumination was used to avoid stimulating the visually-evoked responses. The LED was approximately 10 mm away from the specimen plate. That distance, as well as the small size of the LED and a wide emitting angle, produced a relatively homogeneous illumination.

The digital eyepiece recorded a video with a resolution of 1024 × 768 pixels at a speed of about 7.8 frames per second. This speed produced more than 20 frames at each rotation cycle with a period of four seconds. The infrared filter inside the digital eyepiece was removed to increase the infrared sensitivity.

### Data acquisition

After the larvae were properly mounted and positioned, the video recording was performed with *ScopePhoto*, the software that accompanied the digital eyepiece. Before stimulation, the recorded frames were used to check the illumination. Ten seconds after the video started, the motor was turned on by the controller software *Motion planner *(Parker Hannifin). During the experiment, the motor moved the platform in a sinusoidal profile of amplitude ± 45°. After a one-minute recording, which included about 13 cycle rotations, the video was saved as a Windows Media Video (WMV) format file for analysis.

During the rotation, the motor controller was also used to control the infrared LED to synchronize the video with the rotation. In each rotation cycle, the controller sent out a 100 ms pulse to the infrared LED when the angle of the motor was at about + 28° in the clockwise direction. The illumination was turned off during the 100 ms pulse. This resulted in a dark frame in the video in every rotation cycle. This dark frame was detected by the image processing program and was used to synchronize the eye movements with the rotation angle changes (see Movie 1 in Additional file 1).

### Image processing

**Figure 2:**
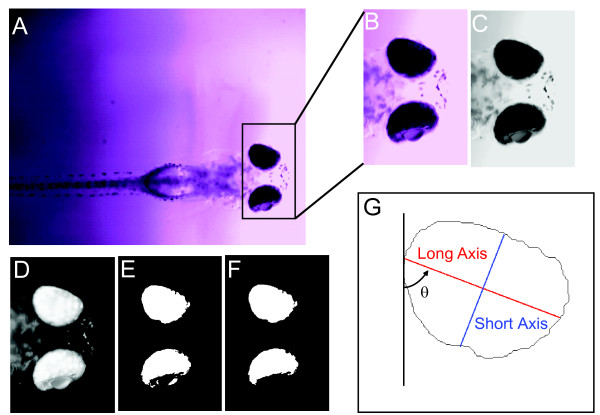
**Defining the eye regions in fish larvae**. Six steps (A-G) were programmed in MATLAB to quantify eye movements/eye changes in the videos. A head region image (B) was first outlined and extracted from the original image frame (A). The head region was converted into a grayscale image (C) with inverted color (D). Then, the inverted grayscale image was converted into a black-white image (E) using an arbitrary threshold. This black-white image was simplified by removing extra punctae around the retina to define the eye more clearly (F). The final step was to calculate the parameters used to quantify changes during eye movements. (G) Features calculated from the extracted eye region. θ designates the rotation angle. The red line indicates the long axis, and the blue line the short axis of the eye.

The recorded video (see Movie 1 in Additional file 1) was processed in MATLAB (Mathworks, Natick, MA, USA) off-line. Figure 2A shows an image frame from a recorded video. The first step was to define the area of the fish head. As shown in Figure 2B, an imaging region containing the fish head was selected manually from the first frame of the video. Since there was no relative motion between the digital eyepiece and the fish, this region was the same for every frame. After defining the head area, a small image portion was cut from each frame. This process substantially reduced the amount of data to process. The colored image was then converted into the grayscale image shown in Figure 2C.

The second step was to define the eyes from the head image, and a grayscale threshold was then applied to invert the grayscale image (Figure 2C) into a black-and-white image (Figure 2D). The threshold was chosen using Otsu's method [18]. This was implemented in MATLAB with the function *graythresh*. Alternatively, manual adjustment of the threshold was sometimes used due to the image intensity change resulting from the motion of the E3 media around the fish head. A scale factor was then applied to the threshold. With a scaled threshold, the area containing the eye was defined for analysis (Figure 2E). An area threshold was then applied to the black-and-white image to remove the small dark island formed by the lens (Figure 2F). The two eyes were then separated in order to calculate the parameters for quantifying the rotations. A similar process was introduced in Beck et al., 2004. Different from that method, here we modified that previous method by confining the eye area to the iris, which is darker than the rest of the eye. This definition of the eye area facilitated the detection of the eye rotation along the anterior-posterior axis, as will be discussed in the next section.

### Quantification of rotation of the eye

After defining the eye region, features were extracted and calculated from the eye to quantify the eye rotation and then to evaluate the reflex. During the experiments, eye rotations on two planes were observable: rotation about the dorsal-ventral axis and rotation about the anterior-posterior axis (see Movie 1 in Additional file 1). The former is on the image plane and the eye angle can be used to quantify it. To measure this angle, the extracted eye region was approximated by an ellipse, and the angle of the long axis was used to represent that of the eye, shown as θ in Figure 2G (outline of upper eye in panel 2F). The eye angle, together with the mass center of the eye, determined the long axis of the eye (red line in Figure 2G). Both the angle and the mass center coordinate were an output of a MATLAB function *regionprop*. The short axis (blue line in Figure 2G) was drawn perpendicular to the long axis. The length of the long axis and short axis were also determined by the function *regionprop*.

Because the eye rotation around the anterior-posterior axis was not in the image plane, direct measurement of this rotation was not practical. The videos showed that the anterior-posterior rotation resulted in a change in the shape of the eye (see Movie 1 in Additional file 1). By measuring the shape change of the eye in each image frame, we could quantify the rotation by examining changes in total area or ratio of the long and short axes.

**Figure 3:**
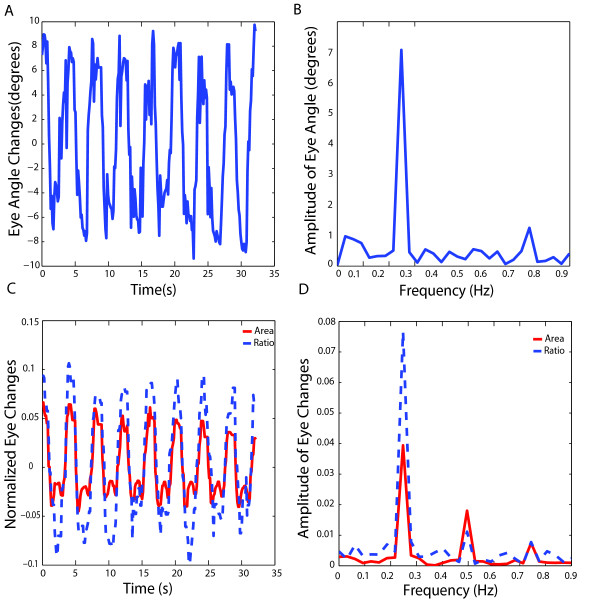
**Features for quantifying eye movements under infrared illumination**. (A) Plot of changes in eye angle of a single eye over time. (B) Amplitude spectra of the continuous waveform in (A) as a function of frequency. A peak at 0.25 Hz corresponded to the rotation period of 4 seconds as shown in (A). In this case, the largest value of angle change observed was about 7 degree. (C) Normalized eye movements derived from changes in the total area (red line) or the long and short axis ratio (blue dashed line) of a single eye. (D) Amplitude spectra of (C). The fraction change at 0.25 Hz is indicated in the brackets. Note that the eye ratio change yielded higher peaks at 0.25 Hz than changes in total eye area in both time domain (C) and spectral (D) plots.

In Figure 3, the results of 8-cycle tests are shown. Figure 2G shows an eye profile and the long axis and short axis of the eye. The eye angle, which is the angle between the long axis and the vertical direction of the image frame, is marked as θ. Figure 3A depicts the change of the angle of an eye during an experiment. Fast-Fourier-Transform (FFT) was applied to the waveform in Figure 3A to calculate the spectrum of the time domain signal, as shown in Figure 3B. The peak change or amplitude at the stimulus frequency was used to quantify the response. Our videos revealed that detecting angle changes in response to a vestibular stimulus was not always feasible, so we turned to the other two features of the eyes: total area versus the ratio of the length of the short axis over that of the long axis. The videos indicated that these two features are closely related. While the eye rotates about the anterior-posterior axis, the iris becomes visually thinner. This results in reduction of the area, which is mostly due to the reduction of the short axis length. Figure 3C (time domain plot) and 3 D (spectra) compare the total area and eye axis ratio changes that occurred during the test. To reduce the influence of specimen variation due to eye shape or original position of the eyes, both the ratio and the area were normalized by its mean value during the test. The normalized value was calculated by

$$\tilde{X}\;=\;\frac{X-mean\left(X\right)}{mean\left(X\right)}$$

where X is either the ratio or the area. As shown in both the time domain plots in Figure 3C and their spectra in Figure 3D, the amplitude of the eye axis ratio change was higher than the total area change. Also, the ratio change was less sensitive to the intensity variation, which influenced both the long axis and short axis in a similar manner. We therefore used ratio changes to determine the amplitude of the vestibular-induced eye movements. One drawback of the ratio method is that the ratio saturates when the rotation angle becomes more than ± 10-20 degrees. However, this limitation did not affect our ability to detect differences among various stages of development or genotypes as seen below.

## Results

### Quantifying vestibular-induced eye movements in zebrafish larvae

**Figure 4:**
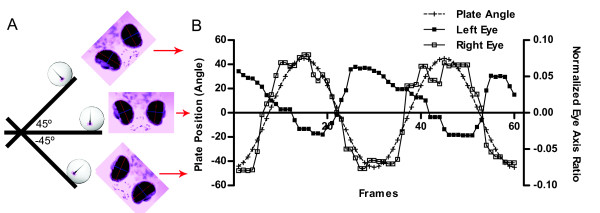
**Representative eye movements in response to platform movements (5 dpf)**. (A) Illustration of the relationship between platform position, larval direction and eye movements. (B) The ratio of long and short axis of both eyes changed sinusoidally. The counter movements of the two eyes followed the platform movements (dashed line). Red arrows between (A) and (B) shows the corresponding position of the larva with respect to platform angle.

To observe eye movements, we rotated larvae on the platform ± 45 degrees at 0.25 Hz. We found that upon stimulation with sinusoidal movements at 0.25 Hz, wild-type larvae at 5 dpf moved their eyes sinusoidally if positioned vertically, with the head pointing downward (Movie 1 in Additional file 1, Figure 4A,B). Larvae also responded if mounted in the opposite direction with the head pointing up (data not shown). In our experiments, larvae were positioned off-axis by 3.2 cm, and movements of the platform lead to a combination of head tilt, and centripetal and tangential acceleration of the specimen (see Appendix A for magnitude of each component). Figure 4B shows the average ratio changes of the long and short axes of both eyes of a representative specimen. In general, the average ratio changes in wild-type larvae displayed a robust response to changes of platform position. We performed our experiments in the dark with infrared illumination to eliminate visual cues. Accordingly, the eye movements we observed in the dark were vestibular-induced rather than visual responses.

**Figure 5:**
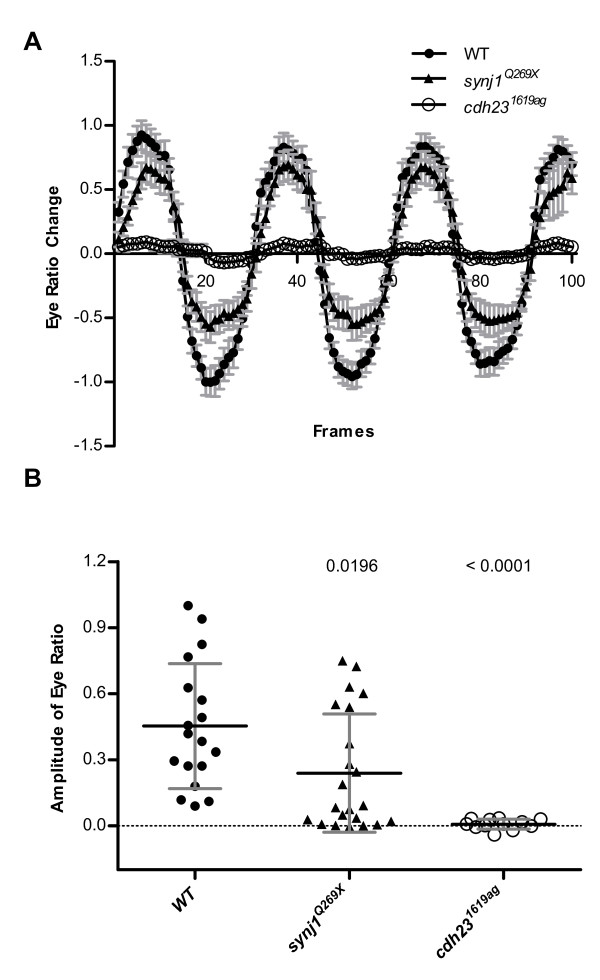
**Vestibular-induced eye movements in wild-type and mutant larvae (5 dpf)**. (A) Averaged eye movements of ***cdh23^1619ag^***, ***synj1^Q269X ^***and wild-type larvae. Mean ± S.E. are depicted. (B) Amplitude of eye movements of ***cdh23^1619ag^***, ***synj1^Q269X ^***and wild-type larvae. The data points shown are the peak amplitude values at the rotational frequency calculated by subtracting the background, which is the average value of other frequencies. Each data point is derived from a single eye (n > 6 fish for each genotype). P values shown in (B) were determined by unpaired two-tailed t-tests of data collected from wild-type versus mutant larvae.

To confirm that eye movements were driven by the vestibular system, we measured the response of auditory/vestibular mutants. Eye movements were measured in *cdh23^1619ag ^*mutants, which lack hair-cell microphonics [8,16], and *synj1^Q269X ^*mutants, which have hair-cell synaptic transmission defects [14]. The *synj1*^Q269X ^mutants showed reduced average eye-ratio changes as previously reported and the *cdh23*^1619ag ^mutants did not have any detectable eye movements during rotation (Figure 5A). To quantify the sinusoidal eye movements, the amplitude of ratio changes of each eye at the rotation frequency was calculated and normalized to the highest value seen with the wild-type larvae in all following figures. In our experiments, all wild-type larvae showed eye movements in the videos, however several had low amplitude values. On occasion, illumination can vary such that the program miscalculates the retina region, resulting in depressions or concave regions within the peak regions and hence reduced calculated amplitude values. However, one can still detect overall differences between wild-type and mutant larvae. As shown in Figure 5B, mutants carrying the *synj1*^Q269X ^allele had a significantly lower mean amplitude of response (0.24 ± 0.27 s.d.; n = 11 larvae) than wild-type larvae (0.45 ± 0.28; n = 9 larvae), and mutants homozygous for the *cdh23*^1619ag ^allele had nearly zero amplitude values (0.01 ± 0.02; n = 6 larvae). Loss or reduction of eye movements in mutants with vestibular defects provided further evidence that we were observing a vestibular response to acceleration of the specimen.

**Figure 6:**
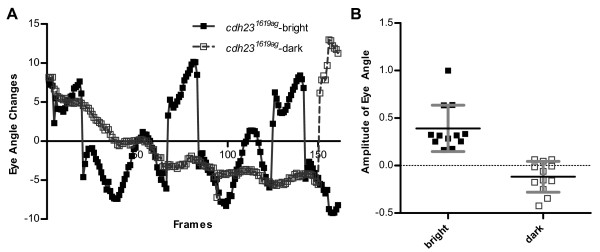
**Presence of the OKR, but not vestibular-induced eye movements in *cdh23^1619ag ^*mutants (5 dpf)**. (A) Representative changes of eye angle from a single ***cdh23^1619ag ^***mutant larva in both dark and bright conditions. With bright illumination, the mutant larva showed changes in eye position in response to platform movements, whereas in the dark, the eye of this mutant larva spontaneously twitched once during the 150**^th ^**frame. (B) Amplitude of eye movements of ***cdh23^1619ag ^***mutants in bright and dark conditions. The same larvae were tested under both conditions (n = 6).

A difference between the OKR observed previously [11,12,19] and the vestibular-evoked response reported here is the nature of eye movements. With respect to OKR responses, zebrafish larvae move their eyes in saccades around the vertical axis in the same plane [12], whereas with vestibular-evoked responses, their eyes rotate around the anterior-posterior axis of the body (see Movie 1 in Additional file 1). In a parallel experiment, the *cdh23^1619ag ^*mutants showed vigorous eye movements in bright light (see Movie 2 in Additional file 2) with typical gaze shifts for visually-evoked responses (Figure 6A). Quantification showed that these eye movements in bright light were driven by platform movements (Figure 6B; 0.39 ± 0.24 for bright light; -0.12 ± 0.16 for dark conditions, n = 6 larvae). This result suggested that *cdh23^1619ag ^*mutants have visual responses but lack vestibular responses. Together, our results confirmed that vestibular-induced eye movements are robust in larvae and can be quantified for comparative studies.

### Earth horizontal versus earth vertical rotations

**Figure 7:**
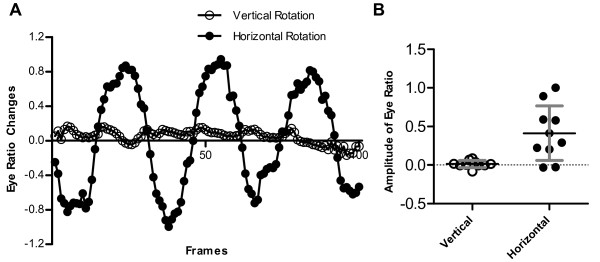
**Vestibular-induced eye movements occur during rotation at 0.25 Hz around an earth horizontal axis, but not around an earth vertical axis**. (A) Representative eye movements of a 5 dpf wild-type larva rotated around an earth vertical axis (empty circles) or an earth horizontal axis (filled circles). (B) Amplitudes of eye movements in the two axes. The same larvae were tested under both conditions (n = 5).

Vestibular-induced eye movements can be evoked by both angular and linear accelerations. The absence of an angular VOR in fish younger than 35 days is most likely due to the morphogenesis and maturation required for the semicircular canals to become fully functional (12). To determine the driving force of the eye movements seen in our experiments, we performed our experiments with the platform in different orientations. To mimic the stimulation used in the experiments by Beck et al., we changed the orientation of our device by 90°. We found that wild-type larvae did not respond to rotation around the earth vertical axis at 0.25 Hz (Figure 7; mean amplitude 0.01 ± 0.05, n = 5). Such a stimulus produces centripetal and tangential acceleration, but no changes with respect to head tilt. These same larvae had a robust response if rotated afterwards about the earth horizontal axis (Figure 7; 0.41 ± 0.35, n = 5 larvae). This result suggests that the change in linear acceleration evoked by head tilt was the driving force of the eye movements.

### Zebrafish larvae develop vestibular-induced eye movements at 3dpf

**Figure 8:**
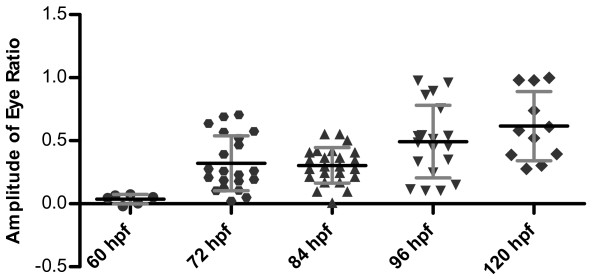
**Development of the vestibular-induced response in zebrafish larvae**. Amplitudes of eye movements of larvae at various developmental stages are shown. The reflex is detectable at 72 hpf and becomes more robust over time. The mean amplitudes (± s.d.) are as follows: 60 hpf, 0.04 ± 0.04 n = 3; 72 hpf, 0.32 ± 0.22 n = 11; 84 hpf, 0.30 ± 0.14 n = 12; 96 hpf, 0.49 ± 0.29 n = 10; 120 hpf, 0.62 ± 0.27 n = 6.

To determine the developmental stage at which zebrafish larvae develop a response to changes in linear acceleration, we measured eye movements of larvae from 60 hpf to 120 hpf. No eye movements were observed in 60 hpf old larvae (Figure 8). The eye movements in response to platform rotation were first detected in 72 hpf fish larvae, and larger eye movements were detected in older fish larvae at 120 hpf (Figure 8). In the videos, the older fish larvae appear to move their eyes more robustly and to a larger degree than younger larvae (data not shown). With respect to the number of fish larvae that have eye movements, we found that at the 72 hpf stage, 90% of the larvae had robust eye movements (n = 11).

### The anterior otolith is required for vestibular-induced eye movements in zebrafish larvae

**Figure 9:**
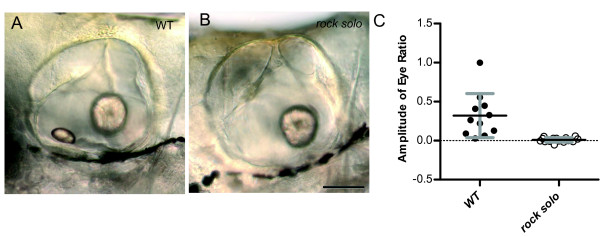
**Vestibular-induced eye movements were absent in larvae lacking anterior/utricular otoliths**. Representative DIC images depicting lateral views of the inner ear of wild-type (A) and ***rock solo ***mutant larvae (B) at 5 dpf. The DIC images were oriented with anterior on the left and posterior on the right. Note the loss of anterior otolith in the mutant, whereas the posterior otolith is unaffected. Scale bar, 100 μm. (C) Amplitudes of eye movements of wild-type siblings and ***rock solo ***mutants.

At 5 dpf, zebrafish larvae have two otoliths that are destined to become the utricular and the saccular otolith. Loss of the anterior/utricular otolith results in balance defects and embryonic lethality [20]. We recently screened for mutants with balance defects and identified an allele that carries a recessive mutation in an unknown gene that we designate as *rock solo*. Larvae homozygous for the *rock solo *mutation do not have anterior otoliths, but the posterior/saccular otolith is still present (Figure 9 A, B). We tested eye movements under infrared illumination in *rock solo *mutants and found that they did not have any detectable responses to rotation about the earth horizontal axis. (Figure 9C; wild-type mean amplitude 0.32 ± 0.28, n = 6; *rock solo *mutant 0.009 ± 0.03, n = 8). In contrast, *rock solo *mutants responded to acoustic stimuli (tapping on the Petri dish) and light touch, suggesting that they have functional sensory hair cells and do not have defects in their motor system (data not shown). This result provides strong evidence that the anterior/utricular otolith in zebrafish larvae is required for vestibular-induced eye movements in response to changes in linear acceleration due to head tilt with respect to gravity.

## Discussion

Our experiments demonstrate that zebrafish larvae have robust eye movements in response to rotation around an earth horizontal axis. At larval stages, both vestibular and visual input may contribute to eye movements. Two lines of evidence support the notion that we are measuring vestibular function rather than visual function. Firstly, motion of the eyes occurred in the dark using infrared illumination. Secondly, vestibular mutants did not respond or had attenuated responses to rotation on the platform. The 1619ag mutation in *cdh23 *used in this study causes a premature truncation of the extracellular domain of Cdh23 [16]. Larvae homozygous for this allele have severe balance defects and lack microphonics, suggesting that mechanotransduction is absent in hair cells [8,16]. Mutant *cdh23^1619ag ^*larvae did not respond to the stimulus under infrared illumination, indicating that hair-cell function was required for movement of the eyes in our experiments. In contrast to experiments in the dark, *cdh23^1619ag ^*larvae exhibited an OKR in response to rotation in bright light, eliminating the possibility that OKRs occurred under infrared illumination. Mutant *synj1 *larvae present the opposite phenotype of *cdh23^1619ag ^*larvae in that *synj1 *mutants exhibit partial vestibular function [14], but vision is lost [21]. We observed that the OKR was absent in *synj1*^Q269X ^mutants (data not shown), indicating that the remaining vestibular-evoked responses were driven by the partially functional vestibular system, and not the visual system. With respect to developmental onset, vestibular-induced eye movements were detectable by 72 hpf. At this stage, zebrafish begin to exhibit OKR responses [11,12] and the auditory/vestibular nerve appears to be fully functional [22]. Our data indicate that the vestibulo-oculomotor projections are operational at this early stage as well.

Testing *rock solo *mutants allowed us to identify which hair cells mediate vestibular-induced eye movements in zebrafish larvae. In every case, the anterior otolith was absent in *rock solo *mutants, whereas the posterior otolith was always present. Mutant *rock solo *larvae failed to respond to earth horizontal rotation of the body, indicating that the anterior utricular macula is required for the response in larvae. In teleosts, the utricular otolith has been previously implicated in vestibular function [6,20] whereas the posterior saccular otolith is thought to be primarily for hearing [23]. Larval zebrafish begin to maintain balance, keeping their dorsal side up, as early as 3 dpf [20]. Following a startle involving sound, touch, or vision, they can coordinate their motor system to produce a forward movement, with an upright posture. Experiments in adult frogs have also shown that the utricular otolith is important for sensing linear acceleration and gravity [24]. Our experiments with *rock solo *mutants support the notion that the anterior otolith acts as a detector of linear acceleration in developing larvae.

The rotation around the earth horizontal axis using our set up presents a complex stimulus to the larval vestibular system. The stimulus includes linear acceleration components of centripetal and tangential acceleration, as well as changes in linear acceleration due to head tilt with respect to gravity. The vestibular system typically uses combined semicircular canal and otolith information to distinguish between translational and roll tilt movements [25]. Both types of inputs should be able to evoke compensatory eye movements [26]. However, we did not observe any eye movements in fish larvae during rotations about an earth-vertical axis. One reason is that the vertical-axis rotation we delivered would primarily stimulate semicircular canals, which are not fully developed in our preparation [12]. A second reason is that the centripetal and tangential accelerations due to the off-axis location of the preparation produced only negligible otolith stimulation (See Additional file 3: appendix A). In contrast, during earth horizontal-axis rotation, there was a large change in linear acceleration that provided a sufficient stimulus to the otoliths. Thus, we infer that the vestibular-induced eye movements we observed in larvae were due to otolith stimulation evoked by the change in head tilt of the specimen. This hypothesis is supported by our experiments with *rock solo *mutants.

The eye movements we observed in larvae included changes in eye position about the dorsal-ventral axis. These movements represent compensatory VOR responses. Other movements include skewed vertical eye movements (about the anterior-posterior axis) and are most likely related to the ocular tilt reaction (OTR) present in lateral-eyed animals such as fish or rabbits (reviewed in 27). In such animals, the OTR is thought to be an otolithic righting reflex. Our measurement of the changes in ratio of eye area included both VOR and OTR movements. Despite the complexity of the eye movement, the vestibular-evoked changes in eye position are sufficiently robust, permitting comparison of responses among mutants and experimental parameters.

## Conclusions

Our results indicate that zebrafish larvae exhibit robust eye movements in response to changes in head tilt with respect to gravity. Our data also confirms that zebrafish larvae rely on the anterior/utricular otolith for maintaining an upright position and coordinating movements with respect to gravity. Measuring the robustness of vestibular-induced eye movements will be invaluable for genetic or pharmacological studies of vestibular function in larvae. In addition, the ability to test vestibular function at earlier stages is especially useful for early lethal phenotypes or accessing gene knockdown with morpholinos as their effectiveness normally decreases over time.

## List of symbols and abbreviations

dpf: days post-fertilization; hpf: hours post-fertilization; FFT: Fast-Fourier-Transform; OKR: optokinetic reflex; VOR: vestibulo-ocular reflex; WMV: Windows Media Video; OTR: ocular tilt reaction.

## Authors' contributions

WM, FC and TN designed experiments, WM and FC performed experiments, AN provided reagents, WM, FC, AN and TN wrote the paper. All authors read and approved the manuscript.

## Supplementary Material

Additional file 1 **Supplementary Movie 1**. Representative eye movements of a 5 dpf wild-type larva rotated around an earth horizontal axis under infrared illumination. Click here for file

Additional file 2 **Supplementary Movie 2**. Eye movements of a homozygous ***cdh23^1619ag ^***larva in dark (upper panel) and bright conditions (lower panel) at 5 dpf. Click here for file

Additional file 3 **Appendix A**. Comparison of accelerations applied to the otolith Click here for file
