# Supplementary material for: Quantification of vestibular-induced eye movements in zebrafish larvae
Source: BMC Neurosci. 2010 Sep 3;11:110. doi: 10.1186/1471-2202-11-110 (PMC2941499; doi:10.1186/1471-2202-11-110)
Supplement: Additional file 3 — Appendix A. Comparison of accelerations applied to the otolith [file 1471-2202-11-110-S3.DOCX]

During our experiments, three different forces were acting on the otoliths: (*i*) gravity; (*ii*) centripetal force; and (*iii*) tangential force. Because the forces were proportional to the mass of the otolith, the acceleration, rather than force, is used in the analysis below. In the following sections, we consider the acceleration of a single otolith.

**Gravity change**

As shown in Figure A1, the anterior macula is at an angle of 23° from the fish body axis. To simplify our calculations, we assume that only the gravity component in the direction parallel to the plane of the anterior macula $g_{Eff}$ (effective gravity) stimulates vestibular hair cells. The gravity does not change during the rotation, but $g_{Eff}$ changes with the rotational angle:

$g_{Eff}=g\cdot cos\left( \theta\right)$ (A1)

where ѳ is the angle of the anterior macula to the vertical axis (gravity). As shown in Figure A1, cycle, the fish body angle changes from -45° to 45° and ѳ changes from 68° to -22° during rotation. The peak-to-peak change of g_Eff_ is $g\cdot cos\left( 0^{\circ} \right)- g\cdot cos\left( -68^{\circ} \right)=0.625g=6.13$(m/s^2^).

(This analysis assumes the anterior macula is perpendicular to the rotation plan, which is also the coronal plane of the fish. In zebrafish, the angle between the anterior macula and the coronal plan is about 76°. Therefore, the maximum change of $g_{Eff}$ is actually 6.13*sin(76°) = 5.95 m/s^2^.)

**Centripetal Acceleration**

Here we estimate the centripetal force on the otolith. The fish was mounted at a radial distance 3.2 cm from the rotational axis. The centripetal acceleration can be calculated by $a_{C}= \omega^{2}\cdot r$, where ω is the angular velocity and r is the radian of the rotation. In the experiment, the fish was moved in a sinusoidal manner. Its position can be described as

$\varphi=\varphi_{0}\cdot cos(\Omega t)$ (A2)

where $\varphi_{0}=\frac{\pi}{4}$ is the maximum rational angle; $\Omega=\frac{2\pi}{T}=\frac{\pi}{2}$ is the angular frequency (in radians/s), T= 4 second is the rotational period. Therefore, the angular velocity

$\omega=\frac{d\varphi}{dt}={-\varphi}_{0}\cdot\Omega\cdot sin(\Omega t)$ (A3)

The maximum angular velocity is $\omega_{m}= \varphi_{0}\cdot\Omega=\frac{\pi}{4}\cdot\frac{\pi}{2}=\frac{\pi^{2}}{8}$, and the maximum centripetal acceleration is $a_{Cm}= \omega_{m}^{2}\cdot r=({\frac{\pi^{2}}{8})}^{2}\cdot0.032=0.049$ (m/s^2^). This value is less than 1/120 of change due to tilt with respect to gravity.

**Tangential Acceleration**

Aside from centripetal acceleration, changes of velocity during rotation apply tangential acceleration to the otolith. The tangential acceleration is in the direction along the tangent of the sinusoidal movements and it can be calculated by$a_{T}=\frac{d\omega}{dt}\cdot r$. With ωcalculated from A3, then the tangential acceleration

$$a_{T}=\frac{d\omega}{dt}\cdot r={-\varphi}_{0}\cdot\Omega^{2}\cdot cos(\Omega t)\cdot r$$

The maximum tangential acceleration is

$$a_{T}={-\varphi}_{0}\cdot\Omega^{2}\cdot cos\left( \Omega t \right)\cdot r=\frac{\pi}{4}\cdot({\frac{\pi}{2})}^{2}\cdot0.032=0.062(m/s^{2})$$

This maximum tangential acceleration is about 1/100 of the change due to tilt with respect to gravity. Therefore, we conclude that the effective gravity change was the major stimulus to the vestibular hair cells, and the effect of the centripetal and tangential forces were negligible in our experiments.


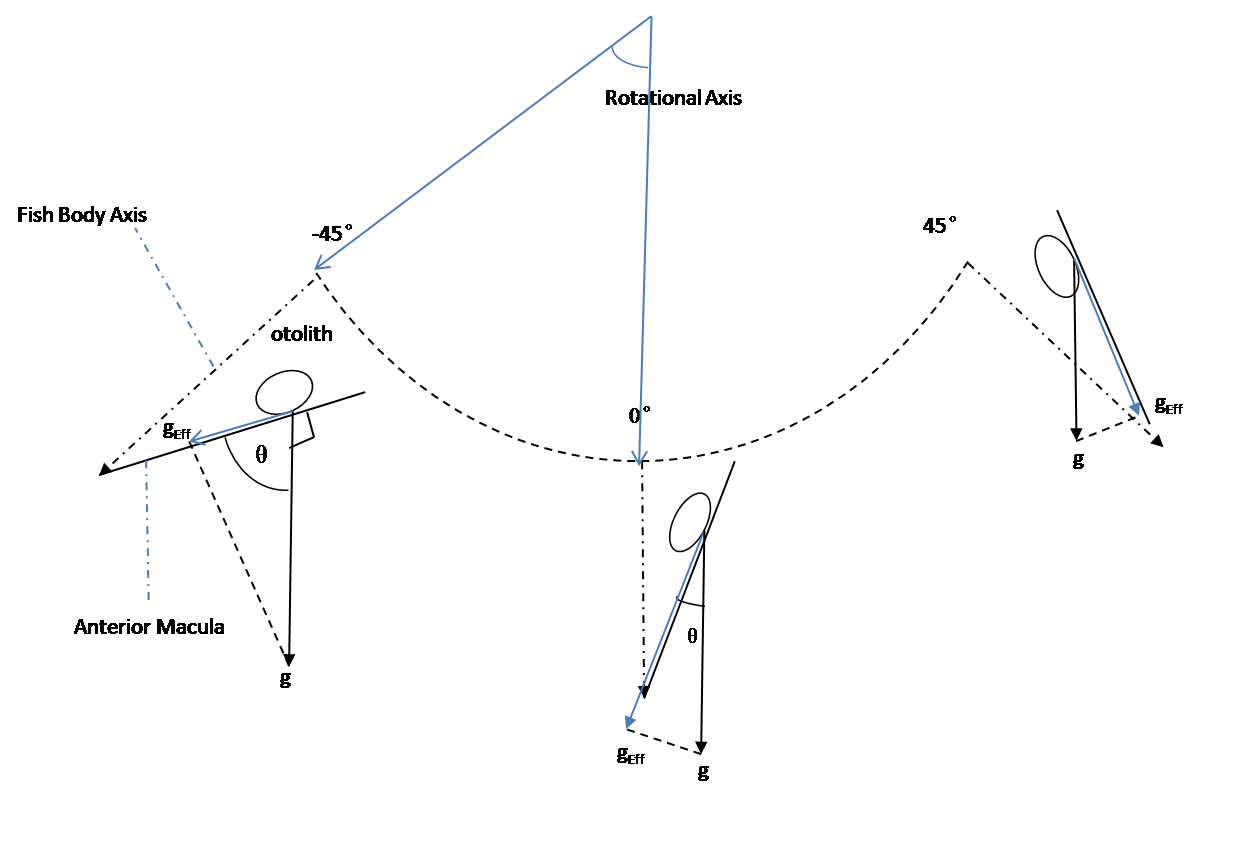


Figure A1. Schematic of g_Eff_ change during a rotation cycle. The relative angular position between the fish body, the anterior macula and gravity is shown in three positions during a rotation cycle. The gravity has a component g_Eff_ in the direction parallel to the plane of the anterior macula. The value of this component is determined by position of the otolith with respect to gravity and the position or angle to the anterior macula ѳ. Therefore, g_Eff_ varies with different rotational position as shown.
